# The crucial role of multiomic approach in cancer research and clinically relevant outcomes

**DOI:** 10.1007/s13167-018-0128-8

**Published:** 2018-02-21

**Authors:** Miaolong Lu, Xianquan Zhan

**Affiliations:** 1 Key Laboratory of Cancer Proteomics of Chinese Ministry of Health, Xiangya Hospital, Central South University, 87 Xiangya Road, Changsha, Hunan 410008 People’s Republic of China; 2 Hunan Engineering Laboratory for Structural Biology and Drug Design, Xiangya Hospital, Central South University, 87 Xiangya Road, Changsha, Hunan 410008 People’s Republic of China; 3 State Local Joint Engineering Laboratory for Anticancer Drugs, Xiangya Hospital, Central South University, 87 Xiangya Road, Changsha, Hunan 410008 People’s Republic of China; 4 The State Key Laboratory of Medical Genetics, Central South University, 88 Xiangya Road, Changsha, Hunan 410008 People’s Republic of China

**Keywords:** Cancer, Multi-omics, Predictive, preventive medicine, Personalization of medical services

## Abstract

Cancer with heavily economic and social burden is the hot point in the field of medical research. Some remarkable achievements have been made; however, the exact mechanisms of tumor initiation and development remain unclear. Cancer is a complex, whole-body disease that involves multiple abnormalities in the levels of DNA, RNA, protein, metabolite and medical imaging. Biological omics including genomics, transcriptomics, proteomics, metabolomics and radiomics aims to systematically understand carcinogenesis in different biological levels, which is driving the shift of cancer research paradigm from single parameter model to multi-parameter systematical model. The rapid development of various omics technologies is driving one to conveniently get multi-omics data, which accelerates predictive, preventive and personalized medicine (PPPM) practice allowing prediction of response with substantially increased accuracy, stratification of particular patients and eventual personalization of medicine. This review article describes the methodology, advances, and clinically relevant outcomes of different “omics” technologies in cancer research, and especially emphasizes the importance and scientific merit of integrating multi-omics in cancer research and clinically relevant outcomes.

## Introduction

**Fig. 1:**
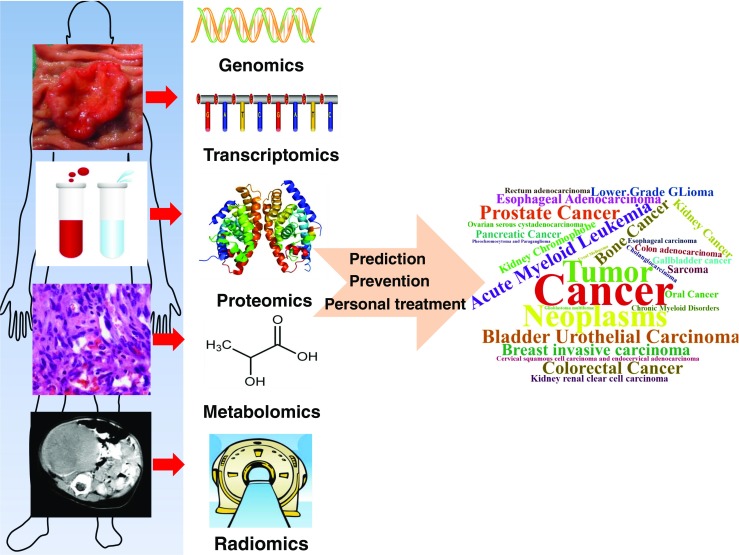
Multiomics and PPPM in cancer

The high-mortality cancer [1] experiences a process of complex and multistep development, malignant cells acquired eight biological capabilities, including sustaining proliferative signaling, evading growth suppressors, resisting cell death, inducing angiogenesis, activating invasion and metastasis, enabling replicative immortality, reprogramming of energy metabolism and evading immune destruction, which are regarded as the hallmarks of cancer [2]. Despite remarkable achievements in cancer research, the exact mechanism of tumor initiation and development still remain unclear yet. Since the Human Genome Project, the emerging scientific era of “omics” has revolutionized the study of cancer [3] (Fig. 1). Omics technologies are primarily aimed at the comprehensive detection of genes (genomics), RNAs (transcriptomics), proteins (proteomics), metabolites (metabolomics), and quantitative features of medical imaging (radiomics) [4]. Omics technologies have a wide-range application in both basic research and clinical treatment of cancer. Based on the next-generation sequencing (NGS), genomics and transcriptomics provide one with a better understanding of the structure of cancer genome and discover differentially expressed genes that drive and maintain tumorigenesis [5–11]. More importantly, this genome profiling has the potential role in establishing different molecular subtypes and stratification of different patients, which is crucial in precisely personalized treatment. High performance liquid-chromatography (HPLC), mass spectrometry (MS), and nuclear magnetic resonance (NMR) technologies are widely used in discovery of new biomarkers and drug targets from cancer proteome and metabolome [12–18]. These biomarkers, including predictive biomarkers for treatment stratification, diagnostic biomarkers for early detection, and prognostic biomarkers for estimation of patient clinical outcome, are important for the predicition and prevention of tumors. At the same time, some key molecules in the pathway and network of tumors such as proteins and metabolites can be recognized as targets for targeted therapy. Currently, varieties of kinase inhibitors have been widely used in targeted therapy of a series of tumors and achieved clinical results. Radiomics is the bridge between medical imaging and personalized medicine. Quantitative analysis of imaging features provides not only the tumor phenotype but the underlying genotype information, which extends the analysis of imaging from qualitative to quantitative analyses and finds the clinical significance that cannot be found with the naked eye. The alterations in the levels of DNA, RNA, protein, metabolite, and medical imaging constructed the myriad of dysfunctionally mutually associated molecular networks making cancer be a complex systems biology disease [19–21]. Any individual study in a level is insufficient to clarify the intricate pathogenesis of a cancer. The integration of multi-omics data plays a pivotal role in elucidation of the molecular mechanism of tumorigenesis and discovery of new biomarkers and drug targets [19, 22]. Thus, a radical shift in cancer treatment is occurring in terms of predictive, preventive, and personalized medicine (PPPM) [23–25]. This review article describes basic principle, challenges, advances and clinical applications of different “omics” technologies, and highlights the significance of integrating multi-omics data in cancer research and in evaluating clinically relevant outcomes.

## Methodology and application of genomics in cancer research and clinically relevant outcomes

### Methodology

Since study found that the abnormal chromosome distribution during cancer cells division suggest a role in malignancy in 1914 [26], ones began to explore the connection between abnormal genetic substance and tumorigenesis. The in-depth studies of chromosome discovered Philadelphia chromosome that was resulted from the translocation between chromosome 9 and 22 in chronic myelogenous leukemia (CML) cells [27]. Since a seminal discovery of a single point mutation of HRAS (a guanosine was substituted to thymidine) that was responsible for the activation of oncogene in T24 human bladder carcinoma cells in 1982 [28], more oncogenes such as EGFR [9], RAS [29], PI3K [30], and ERK [31] have been recognized. Those findings promote scientists to increasingly understand cancers that are derived from accumulation of genomic alternations, including base substitutions, small insertions and deletions, chromosomal rearrangements and copy number alterations and microbial infections [32]. Less than 3 years after the completion of Human Genome Projects, the National Institutes of Health has officially launched the pilot stage of an effort to create a comprehensive catalogue of the genomic changes related to cancer in 2006, namely the Cancer Genome Atlas (TCGA) [33]. Moreover, the international Cancer Genome Consortium (ICGC) and the Cancer Genome Project of the United Kingdom share the same goals that identify all genomic alternations significantly associated with cancer.

The development of cancer genomics is inseparable from the progress of DNA sequencing technology. From the first-generation sequencing to the next-generation sequencing, DNA sequencing technology has developed by leaps and bounds. Here, the development of technologies in DNA sequencing is reviewed.

First, Sanger invented “the dideoxy method” in 1977 [34], which improved the method of the previous “plus and minus” [35, 36] for DNA sequencing. Sanger sequencing based on the selective incorporation of chain-terminating dideoxynucleotides by DNA polymerase during *in vitro* DNA replication had been predominant method in this filed for almost 30 years [34, 37]. With long read lengths (up to ~ 1000 bp) and high per-base “raw” accuracies as high as 99.999% [38], Sanger sequencing achieved a number of monumental accomplishments, including completing of the Human Genome Project [37]. However, it has the obvious disadvantages of high cost and low throughput [3, 37]. The demand for entirely new technologies that deliver fast, inexpensive, and accurate genome information catalyzed the development of next-generation sequencing (NGS) technologies.

The second-and third-generation technologies are referred to as NGS [37]. By now, several commercially available platforms such as Roche/454, Illumina/Solixa, Life/APG, and Helicos BioSciences are all characterized by cyclic array sequencing summarized as the sequencing of a dense array of DNA features by iterative cycles of enzymatic manipulation and imaging-based data collection [38]. Parameters of partial platforms were summarized (Table 1). The advantages of second-generation sequencing relative to Sanger sequencing include the higher speed and throughput, cyclic array sequencing to provide with > 10^6^ reads/per-array and lower cost, the relatively easier gene library construction, higher degree of parallelism, and more efficient use of reagents [38, 39]. The disadvantage that limited the application of these platforms are shorter read lengths with an average read length range from 32 to 330 bp [37]), which creates challenges for genome alignment and assemble [3, 37, 38, 40, 41]. In the aspect of raw accuracy, the NGS platforms are at least tenfold less accurate than Sanger sequencing [38]. In addition, the overall cost is still high, 1–60 dollar/megabase [38], although the cost per base is lower by several orders of magnitude compared to Sanger sequencing [39].

**Table 1:** Parameters of partial platforms

Platform	Method	Read length (bp)	Throughput	Reads	Runtime
SOLiD 5500xl	Sequencing by ligation	2 × 60	95 Gb	800 M	6 d
SOLiD 5500xl Wildfire		2 × 50	240 Gb	2.4 B	10 d
Illumina HiSeq2500 HT v3	Sequencing by synthesis (cyclic reversible termination)	2 × 100	600 Gb	3 B	11 d
Illumina HiSeq2500 HT v4		2 × 125	1 Tb	4 B	6 d
454 GS Junior	Sequencing by synthesis (single-nucleotide addition)	Up to 700	35 Mb	0.1 M	10 h
454 GS FLX Tianium XL+		Up to 1000	700 Mb	~ 1 M	23 h
Pacific BioSciences RSII	Single molecule real time long reads (phospholinked fluorescent nucleotides)	10–15 Kb	500 Mb–1 Gb	~55,000 K	4 h
Oxford Nanopore MK1 MinlON	Single molecule real time long reads (phospholinked fluorescent nucleotides)	Up to 200 Kb	Up to 1.5 Gb	> 100,000 K	Up to 48 h

The third generation of sequencing technology such as PacBio RS and Oxford Nanopore sequencing is developed to solve the shortcomings of the second-generation [42], with fundamental feature of the single molecule sequencing but not requirement of any PCR process, which effectively avoids the PCR bias caused by the system error, improve the read length, and maintain the advantages of high-throughput and low cost of the second-generation technology.

### Application

All cancers arise as a result of changes that have occurred in the DNA sequence of the genomes of cancer cells [43]. Thus, discovery of new somatic mutations, especially the “driver gene” mutations, has been at the heart of cancer research for more than a century. With the application of the NGS, identification of all genomic abnormalities in cancers has been turned from fantasy into reality. TCGA research network has showed the comprehensive genomic characterization of squamous cell lung cancers [44], gastric adenocarcinoma [45], human colon and rectal cancer [46], human glioblastoma [47], and ovarian carcinoma [48]. The study of lung squamous cell carcinoma (LSCC) found a mean of 360 exonic mutations, 165 genomic rearrangements, and 323 segments of copy number alteration per tumor, and loss-of-function mutations that are not reported previously. Besides, a potential therapeutic target was identified to offer new avenues of investigating the treatment of LSCCs [44]. Up to date, many types of cancers have been sequenced with whole genome sequencing (WGS) or targeted genome sequencing (Table 2) [7, 49–58].

**Table 2:** Examples of the application of NGS in cancer research

Author and published data	Cancer	Sample source	The number of sequencing sample	Platform	The significant of result in PPPM
Marchetti et al. 2014 [49]	Non-small-cell lung cancer (NSCLC)	DNA from blood circulating tumor cells (CTCs)	59 (37 NSCLC with EGFR mutation, 10 breast cancer without EGFR mutation and 12 healthy donors)	Roche 454 GS junior	Analysis of CTCs based on CellSearch System and NGS is a reliable method to detect EGFR mutation, which have important significance in stratifying patients
Vignot et al. 2013 [7]	NSCLC	DNA from archived surgical samples	30 (15 pairs of primary matched metastatic tumor tissues)	HiSeq2000 (Illumina, San Diego, CA)	Genomic somatic alternations of primary tumor tissue may provide much of the relevant information required to guide treatment on recurrence
Hagemann et al. 2014 [50]	NSCLC	DNA from formalin-fixed, paraffin-embedded (FFPE) tumor tissue	209 (147 adenocarcinoma, 4 large cell neuroendocrine, 9 poorly differentiated, 6 sarcomatoid, 36 squamous cells)	Illumina HiSeq 2000, MiSeq, HiSeq 2500	Based on NGS well-chosen FFPE tissue can provide relevant genomic information such as potential actionable mutations
Beltran et al. 2012 [51]	Advanced prostate cancer (PCa)	DNA from formalin-fixed, paraffin-embedded (FFPE) tumor tissue	45 (25 metastatic castration resistant PCa, 4 metastatic hormone-naive PCas, and 16 primary localized PCas)	HiSeq2000® (Illumina-Solexa)	Based on NGS, comprehensively genomics information derived from FFPE tissue has the potential to select appropriate targeted therapy patients, discover new biomarkers, drug targets
Berger et al. 2011 [52]	PCa	DNA from tumor tissue	14 (7 tumor/normal tissue pairs)	Illumina GA II sequencer	The first whole genome sequencing analysis of human prostate cancer promising to establish genomics criterion to stratify patients, uncover mechanisms of carcinogenesis and identifies novel targets for therapeutic intervention
Weisman et al. 2016 [53]	Breast cancer	DNA from triple negative breast cancer tissue	78 (39 tumor/normal tissue pairs)	HiSeq2000® (Illumina-Solexa)	This study identified the triple negative breast cancers with apocrine differentiation as a distinct subset, which elevate the precision treatment of triple negative breast cancer
Janku et al. 2014 [54]	Hepatocelluar carcinoma(HCC)	DNA from archived surgical samples	14 (4 liver biopsy, 3 liver resection, 1 liver transplant, 4 metastatic lesion, 2 not available)	HiSeq2000® (Illumina-Solexa)	This study provide a comprehensive genomic profiling of advanced HCC and the result of targeted therapy and highlight the important role of NGS based genomics in cancer research
Ross et al. 2014 [55]	Intrahepatic cholangiocarcinomas (ICC)	DNA from formalin-fixed, paraffin-embedded (FFPE) tumor tissue	28 (16 liver biopsies, 10 liver resections, 1 in lymph node metastasis, 1 in lung metastasis)	(Illumina HiSEquation 2000 (Illumina Inc., San Diego, CA)	This study provide a comprehensive genomic profiling of ICC, in which genomic alternations have the potential to determine the personal therapies and discover novel druggable target
Ward et al. 2016 [56]	Bladder cancer	DNA from urine cell pellets	231 (120 primary bladder cancer, 20 non-cancer, 91 bladder cancer patients post-TURBT)	Illumina MiSeq	This non-invasion method detecting reported bladder cancer mutations based on sequencing of DNA from urine cell pellets has 70% sensitivity and 97% specificity
Liang et al. 2012 [57]	Pancreatic adenocarcinoma (PA)	DNA from tumor tissue and peripheral blood mononuclear cells (control)	6 (3 paired tumor/normal samples)	Illumina HiSeq 2000	The whole genome sequencing generated comprehensive genomic information of 3 PA patients provide individually potential tumorigenic mechanisms and visibe therapeutic targets
Kim et al. 2014 [58]	Bladder cancer	DNA from tumor tissue and peripheral blood mononuclear cells (control)	218 (109 patients with tumor tissue and germline blood)	Illumina HiSeq 2000/2500	This study demonstrated the relationship between genomic mutations and treatment outcomes, and genomic markers can guide personal treatment and elevate the therapy efficiency

The application of high-speed and high-throughout NGS technologies improves significantly the analysis of cancer genome, and reveals the full repertoire of mutated cancer genes, which not only can be used to guide the discovery of new targeted drugs, but also have an overwhelming impact on understanding of cancer biology and accelerate strategies in PPPM in cancer. For example, gene fusions resulting from chromosome translocations have an important role in the initial steps of tumorigenesis with evidence of discovery of gene fusions in all malignancies [59]. Functionally recurrent gene fusions provide more precisely clinical-related subclassifications of traditionaly morphological classification of tumors and accelerate the development of specific targeted therapies. Previously, because of lacking systematic approaches, this type of molecular abnormality has been regarded as a fundamental mechanism in haematological and soft-tissue malignancies. Recent years, with the application of NGS, novel recurrent chromosomal rearrangements have been discovered in many kinds of solid tumors, such as TMPRSS2-ETS fusion oncogenes in prostate cancer (Pca) [60], EML4-ALK fusion oncogenes in non-small cell lung cancer (NSCLC) [61], ETV6-NTRK3 fusion oncogenes in secretory breast cancer [62], BRAF and RAF1 fusion oncogenes in melanoma [63], BRAF gene fusions in pilocytic astrocytomas, pancreatic acinar and papillary thyroid cancers [64]. By July 2017, the Tumor Fusion Gene Data Portal (http://www.tumorfusions.org/) has presented 33 tumor types and a total of 20731 fusion genes information. The common fusion genes are kinase and transcription factors, which play an important role in tumorigenesis and metastasis and shed light on the PPPM practice in cancer [65]. Some clinical studies have evaluated the diagnostic and prognostic values of TMPRSS2-ERG gene fusion for Pca, which demonstrated that TMPRSS2-ERG had prognostic value and its combination with prostate cancer antigen 3 (PAC3) can increase the precision of PSA-based diagnosis [66, 67]. More importantly, the character that TMPRSS2-ERG gene fusion could be measured in the urine makes it an ideal biomarker supplementing the PSA test [67, 68]. ETV6-NTRK3 fusion oncogene was discovered in 90% secretory breast carcinoma (SBC), a rare subtype of infiltrating ductal carcinoma, but not in other ductal carcinomas [62]. In addition, ETV6-NTRK3 fusion oncogene was also reported in a rare salivary gland tumor similar to SBC leading to a newly described type of salivary carcinoma-secretory carcinoma (SC) [69]. Studies demonstrated that ETV6-NTRK3, a chimeric protein tyrosine kinase, depended on insulin-like growth factor 1 receptor signaling and induced insulin-receptor substrate-1 (IRS-1) constitutively tyrosine phosphorylated and consequently activated Ras-Erk1/2 and PI3K-AKT signaling pathways during transformations [70, 71]. Functional studies suggest these cells and cancers may sensitive to kinase inhibitors. A pan-NTRK as well as ALK and ROS1 tyrosine kinase inhibitor, entrectinib, has been found useful in treating a single patient with SC, which demonstrated the potential role of kinase inhibitor in treating of ETV6-NTRK3 fusion gene-associated cancers [72]. EGFR mutants were the most common genomic alteration underlying NSCLC, and patients with EGFR mutants were routinely treated with EGFR kinase inhibitor. Recent years, new recurrent fusion oncogenes EML4-ALK and FGFR3-TACC3 have been identified in NSCLC [61, 73]. These forms of molecular abnormalities have distinct mechanisms of tumorigenesis from EGFR mutants. The former is sensitive to ALK tyrosine kinase inhibitors such as crizotinib (approved by FDA in 2011) and the latter to fibroblast growth factor receptor (FGFR) kinase inhibitors such as BGJ398 (under clinical trials) [73, 74]. These findings complement the genotyping diagnosis of NSCLC and will benefit specific types of patients, ultimately enabling personalized medical treatment.

## Methodology and application of transcriptomics in cancer research and clinically relevant outcomes

### Methodology

The genetic central rule shows that genetic information is transferred from DNA to protein through RNA (mRNA) under precise regulation. The mRNA is regarded as a “bridge” in the process of biological information transfer from DNA to protein. Transcriptome is whole intracellular transcripts and their quantity in a given time and environmental condition. Transcriptome is an essential objective to address the functions of genome, uncover the molecular constituents of cells, and reflect the occurrence and development of a disease. The key aims of transcriptomics are to catalogue all species of transcripts, denote the transcriptional structure of gene, and quantify the expression level of each transcript during development and under different conditions [75]. Unlike genome that is a relatively static entity, transcriptome is dynamic, and modulated by external and internal factors. Therefore, transcriptome serves as a dynamic link between an organism’s genome and its phenotype characteristics [76].

**Fig. 2:**
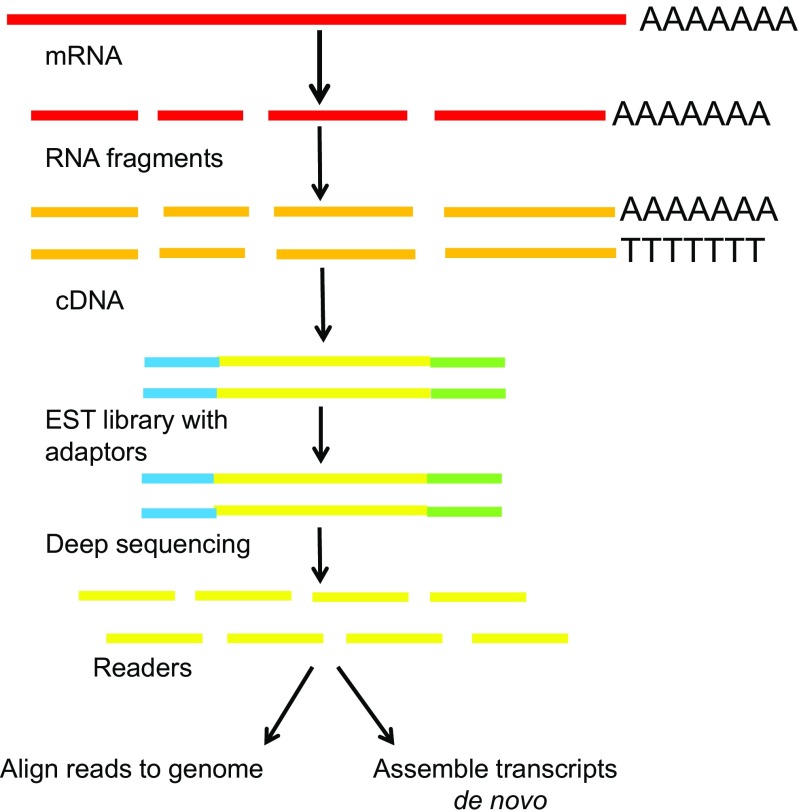
The general workflow of RNA-seq. EST: expressed sequence tag

Up to now, various methods have been developed to study transcriptome, including hybridization-or sequence-based approaches [75]. The former is based on hybridization between nucleic acids, which typically involves incubation of fluorescently labeled-cDNA derived from reverse transcription of different mRNAs with microarrays that are consisted of genes of interest, followed by digitalization with the specialized scanner and image analysis. Information is achieved such as gene name, clone identifier, and intensity values [77]. Recently, tiling microarrays derived from the standard gene expression microarray are composed of oligonucleotide probes that span the entire genome of an organism to provide a more unbiased view of the transcriptional activities within a genome [78]. However, several shortages of these methods include the reliance on existing knowledge of genome sequence, high background levels owing to cross-hybridization, and a limited dynamic range of detection due to both background and saturation of signals. Sequence-based approaches determine cDNA sequence but not rely on the probes. The sequences of cDNA or EST libraries were initially detected by Sanger sequencing approach; however, it is relatively expensive, low throughput, and generally no quantitative information. Afterwards, tag-based methods were developed to overcome those limitations, including serial analysis of gene expression (SAGE), cap analysis of gene expression (CAGE), and massively parallel signature sequencing (MPSS), which can provide high throughput, and precise gene expression levels, but are still based on Sanger sequencing technology that results in an analysis of only a portion of the transcripts and indistinguishing isoforms. The emergence and development of NGS provides a new approach, RNA-seq, for this high-throughput DNA sequencing technique in mapping and quantifying transcriptome (Fig. 2). The advantages of RNA-Seq include (1) high throughput, namely RNA-seq can achieve several to hundred billion of base sequences, which can cover the entire genome or transcriptome; (2) high sensitivity, namely RNA-seq can detect only a few copies of rare transcripts in a cell; (3) high resolution, namely RNA-Seq can achieve single-base resolution with good accuracy and avoid the level of high background; and (4) no reconstructions, namely RNA-seq can be used for the analysis of whole transcriptome of any species, including detection of unknown genes or transcripts, and accurate identification of the cleavage site, and a variable SNP or UTR region.

### Application

Alternative splicing of precursor messenger RNA from a single gene was first discovered about 30 years ago, which produces multiple different functional messenger RNAs, and the corresponding proteins derived from the a single gene [79]. Splicing abnormalities are a common characteristics of cancer [80], occurring in every category of cancer hallmarks [81]. Abnormal splicing could result in aberrant protein variants to involve different functions such as transcription factors, cell signal transducers, and components of the extracellular matrix [82]. The nature of the altered gene products is usually consistent with an active role in cancer. RNA-seq can directly and readily detect RNA splicing events relative to standard gene expression microarray, so it is a power tool in discovering cancer-related alternative splicing, which might be a diagnostic or prognostic marker and potential personalized therapy target.

In the research of NSCLC, a comprehensive study of prognosis-related alternative mRNA splicing using RNA-seq data identified a large number of alternative splicing events that are associated with the prognosis of NSCLC. Furthermore, prognostic predictors based on alternative splicing events were established for risk stratification with excellent performance [83]. RNA-seq also allows quantitative study of alternative splicing. Owing to alternative splicing, the insulin receptor has two isoforms: insulin receptor isoform A (IR-A) and insulin receptor isoform B (IR-B) [84]. Another study used bioinformatics methods to analyze RNA-seq data of both isoforms found that downregulated IR-B level and increased IR-A/IR-B mRNA ratio correlated with lower epithelial-mesenchymal transition and longer survival time. In addition, this phenomenon has been found in other 18 types of cancers, which suggests this ratio could be used as a marker of prognosis and treatment response assessment [85]. In breast cancer, several EMT-associated alternative splicing events have been identified and most of these alternative splicings are regulated by one or more members of splicing factor classes such as PBFOX and ESRP, which may provide new diagnostic and prognostic markers and personalized treatment targets of a breast cancer [86].

Compared to the analysis of DNA sequencing-based structural variations, transcriptomics can provide with an analysis of DNA functional characteristics in the RNA level to link the gene structural feature to its functions and easier discover the causal of physiological or pathological conditions [87, 88]. RNA-seq has been proved to be a useful tool for the discovery of new gene fusions in cancer transcriptome. For example, one rather common and tumor-specific novel fusion gene SYT8/TNNI2 was discovered in analysis of three bladder carcinomas with high-throughput RNA-seq, which has potential clinical relevance [89]. Also, oncogenic gene fusions were revealed systematically in primary colon cancer with IIumina RNA-seq, with a result of a relevant gene fusion occurring 2.5% of all specimens; of them, USP9X-ERAS formed by chromothripsis was considered as highly oncogenic, with the ability to activate AKT signaling [90]. The analysis of ovarian cancer RNA-seq data with a novel computational method for fusion discovery—deFuse provides the first gene fusion discovery of ovarian cancer, which may contribute to the study of tumor initiation, development and treatment [91].

Micro RNAs are short (~ 22 nucleotides in length) non-coding RNAs (ncRNAs) that regulate gene expressions by binding to specific mRNA targets and promoting their degradation and/or translational inhibition [92]. Recent studies suggest that miRNAs play roles in cancer [93–97]. RNA-seq is a powerful tool to uncover unannotated ncRNA species. The abundant expression of miRNA-1323 and its distinct association in tumors arising from a cirrhotic background were discovered in hepatocellular carcinomas (HCCs) [98], and overexpression of miRNA-1323 in cirrhotic-HCCs was correlated with poorer disease-free and overall survivals of patients. In the study of myelodysplatic syndromes, the analysis of RNA-seq data demonstrated that the expression of miRNA was associated with the progression of the disease [99]. The miRNA-mRNA regulatory network was studied in peripheral blood mononuclear cells of small cell osteosarcoma (SCO) with RNA-seq [100], which identified 37 dysregulated miRNA (27 upregulated and 10 downregulated) and 1636 dysregulated mRNAs (555 upregulated and 1081 downregulated), two important signaling pathways including mTOR signaling and cell cycle signaling, and dysregulation of three miRNAs (has-miR-26b-5p, has-miR-221-3p, and has-miR-125b-2-3p) that might be involved in SCO tumorigenesis.

In addition to miRNAs, a large proportion in a transcriptome is long ncRNAs (lncRNAs) with longer than 200 nucleotides, which are often polyadenylated and are devoid of evident open reading frames these [101]. Studies demonstrate that lncRNAs are able to regulate gene expressions at the levels of chromatin modification, transcription, and post-transcriptional processing [101, 102], especially in some human cancers with tissue-specific expressions [103], demonstrating their potential roles in both oncogenic and tumor-suppressive pathways [104, 105]. Currently, the study of lncRNAs is still in its initial stage with studies of only a small part of lncRNAs such as HOTAIR [102, 106], and MALAT1 [107, 108]. However, IncRNAs demonstrate its big potential in PPPM practice, and RNA-Seq is maximizing the coverage of cancer-related lncRNAs in a transcriptome. For example, among 121 unannotated prostate cancer-associated ncRNA transcripts, PCAT-1 was discovered as a prostate specific regulator of cell proliferation and a transcriptional repressor in a subset of prostate patients [109]. RNA-seq systematically identified quintuple-negative lung adenocarcinoma-related IncRNAs [110], including 90 upregulated and 153 downregulated lncRNA transcripts. The functions of 14 predicted lncRNAs such as vasculature development and cell cycle are closely related to the process of cancer development. Another study [111] identified a signature of five lncRNAs (CYP4F26P, RP11-108M12.3, RP11-38M8.1, RP11-54H7.4 and ZNF503-AS1), which might act as an independent prognostic indicator for LUSC with RNA-seq data from TCGA. Similarly, a signature of eight lncRNAs was identified to stratify and predict survival in esophageal cancer [112].

## Methodology and application of proteomics in cancer research and clinically relevant outcomes

### Methodology

Proteins are the effectors of DNAs in a biological system, and the expression levels of all proteins in a proteome would inarguably provide the most relevant phenotype characteristics of that biological system [113]. The goal of proteomics is to characterize information flow with protein pathways and networks to eventually understand the function relevance of proteins in a cell or organism [4]. The proteome has many unique features that distinguish from other omics approaches, and is much more complex than genome and transcriptome. The number of human proteins and their variants or protein species is estimated up to over billions [19]. Also, one gene corresponds to multiple proteins, namely one gene-multiple proteins model but not one gene-one protein model [114, 115]. In addition, variations in a proteome are more measureable than variations in genome and transcriptome [116]. It seems that genome contains all information; however, except for the sequence and copy number of DNAs and RNAs, other information in a genome is difficultly measured with current technologies. Proteome as an important component of a phenome is the final performer of genome functions; much information in a proteome is measurable such as amino acid sequence, splicing, copy number, post-translaitonal modifications (PTMs), variants, spatial conformation, and spatial re-distribution. In the last decade, numerous proteomics studies have focused on protein profiling and protein expression alternations that associate different given conditions.

**Fig. 3:**
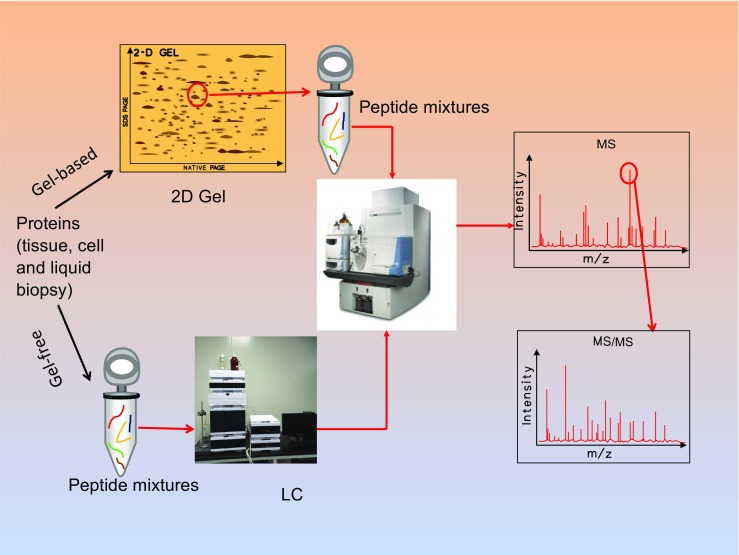
The MS-based proteomics workflow. 2DGE: two-dimensional gel electrophoresis; MS: mass spectrometry; MS/MS: tandem mass spectrometry; and LC: liquid chromatography

Proteomics method commonly includes protein preparation, separation, and identification (Fig. 3). Protein separation is to reduce the complexity of the proteome sample, mainly includes gel- and liquid chromatography (LC)-based approaches. The gel methods include one-dimensional gel electrophoresis (1DGE), two-dimensional gel electrophoresis (2DGE) [117], and two-dimensional difference in gel electrophoresis (2D-DIGE) [118]. The specific antibody must be used in combination with those gel methods if variants of a given protein [118], or a kind of PTM [119–121] need to be detected. The LC methods as proteomic separation technique are extensively used in the field of current proteomics, mainly include 2DLC and multi-dimensional LC (MDLC), and a stable isotope (e.g. iTRAQ and TMT) labeling coupled with 2DLC can quantify the component of a proteome. Moreover, some LC methods in combination with MS are developed to identify protein variants, and protein species [122–126]. MS is the key protein identification technique, which can determine amino acid sequence of a protein [115], and PTM-sites [120]. Different types of mass spectrometers are commercially available, including matrix-assisted laser desorption ionization-time of flight-time of flight (MALDI-TOF-TOF)[127], Fourier transform ion cyclotron resonance (FTICR) [128, 129], triple TOF 5600 or 6600 systems [130], and LTQ orbitrap system [131, 132] with different ion fragmentation models such as collision induced dissociation (CID) [133], electron transfer dissociation (ETD) [134], and electro capture dissociation (ECD) [128, 135], which provides the optimal strategies to identify protein expressions, PTMs, protein variants and protein species. However, one must realize that each mass spectrometer has its own sensitivity and resolute capability, an enrichment strategy is needed prior to MS in analysis of low abundance protein, PTMs, or protein variants [126, 136].

MS-based proteomics includes top-down and bottom-up approaches. Top-down proteomics is able to identify and quantify unique proteoforms through feeding intact full proteins directly into MS, which is capable of providing distinct characteristics of each kind of proteoform with more precise and more abundant biological information [137]. Bottom-up proteomics digests firstly protein components with enzyme, followed by LC fractions and MS-identification, which is able to identify and quantify proteins expressed differentially, and PTMs [138]. Recently, middle-down method that combined top-down and bottom-up strategies receives attentions in that this method not only can avoid redundant peptides sequences but also can analyze large protein fragments [139].

Quantitative proteomics plays very important roles in understanding the biological significance, mainly including 2DGE-based quantitative methods [140, 141], stable isotope-labeled quantitative methods such as isobaric tags for relative and absolute quantification (iTRAQ) [142, 143], and label-free quantitative methods [144, 145] such as selected/multiple reaction monitoring (SRM/MRM) [146, 147], and sequential window acquisition of all theoretical mass spectra (SWATH) [148, 149]. Furthermore, structural proteomics benefits in-depth understanding of the biological functions of a protein in a biological system [150, 151].

### Application

Discovery of new tumor biomarkers is the hot point in the field of cancer research with high-throughput MS-based proteomics. For example, glycosylated proteins represented 50% of the secreted proteome and abnormal glycosylation of proteins has been implicated to play a critical role in cancerous progression [152]. Since more than half of the proven cancer biomarkers are glycosylated proteins, MS-based glycoproteomics can analyze qualitatively and quantitatively thousands of glycosylated proteins with detailed information, which shows a great potential in discovery of novel cancer biomarkers. Thus, glycoproteomics has extensively used in cancer research. Several examples are taken here.

Quantitative proteomics analysis of fucosylated glycoproteins in small cell lung cancer (SCLC) patients [153] found a significant decrease of PON1 protein expressions in the sera of SCLC patients, but a significant increase of PON1 fucosylation. The altered fucosylated glycan patterns and levels of PON1 were used as potential diagnostic and prognostic biomarkers for SCLC. Another MS-based glycoproteomics identified the significantly increased fucosylated haptoglobin (HP) with three α-2, 6-linked sialic acids, in serum of each subtype of lung cancers (19 lung adenocarcinoma, 8 LSCC, 11 SCLC and 7 unknown types) relative to controls [154]. This specific glycan of Hp from the serum can serve as a potential diagnostic glycobiomarker for lung cancer.

Glycoprotein biomarkers were also studied in HCCs. Compared to liver cirrhosis patients, an integrated approach analyzing glycoproteins and their glycosylations in HCC sera found the significantly increased levels of 5 fucosylated glycoproteins, which can be regarded as early diagnostic biomarker candidates with excellent performance [155]. Also, AFP-L3, which is an isoform of AFP, and binds strongly to lens culinaris agglutinin (LCA) by an additional α1-6 fucose residue at the reducing terminus of N-acetylgucosamine, has been determined as an early and highly specific biomarker for HCC with sensitivity 56% and specificity 95% [156].

Quantitative glycoproteomics has been used to study Pca with a high incidence and low mortality [157–159]. Prostate-specific antigen (PSA) was an FDA approved serum biomarker for Pca diagnosis and prognosis with low specificity, and cannot distinguish aggressive Pca from non-aggressive Pca, which might result in overtreatment of non-aggressive Pca patients. To obtain the urgently needed novel biomarker for Pca patients, SWATH-based glycoproteomics discovered and validated two glycoproteins (N-acylethanolamine acid amidase, and protein tyrosine kinase 7) in Pca tissues as Pca aggressive biomarkers [160], which provides a basis for the precise treatment of Pca patients, and reduces side effects of Pca overtreatment.

In addition to glycosylation of proteins, other types of PTMs in proteins also constitute a large number of diagnostic and prognostic biomarker candidates. For example, phosphoprotein secretomics studies provided a set of novel breast cancer subtype specific phosphopeptide candidates in plasma [161]. PGRMC1 is a membrane-related progesterone receptor and an important biomarker for breast cancer progression. Since phosphorylated PGRMC1 will active a series of intracellular signaling, it is a potential therapeutic target [162]. Based on tissue phosphoproteomics method in NSCLCs, PTRF/cavin-1 and MIF have been regarded as new potential biomarkers [163]. Protein tyrosine nitration is another important PTM, which changes the chemical properties of that tyrosine residue and protein functions [151, 164]. 2DGE-based nitroproteomics [119] identified 18 nitroproteins and 20 nitrotyrosine sites in human high-grade astrocytomas, which are associated with a series of biological processes such as drug assistance and signal transduction, provide new insights into pathogenesis of astrocytomas, and benefit the discovery of new biomarkers for its early diagnosis and effective therapeutic targets [165].

Besides biomarkers, proteomics approach is also a guiding tool for the discovery of more potential therapeutic targets, for example, BIRC6 in colon cancer stem cells [166], bone marrow stromal antigen 2 and cyclophilin A in endometrial cancers [167, 168], phosphoglycerate mutase 1 in HCCs [169], anaplastic lymphoma kinase in ovarian cancer [170], and hypusination of eukaryotic initiation factor 5A in BCR-ABL-positive leukemias [171].

Above examples are only windows for the use of proteomics in cancer research. Here, one must realize that the initiation and development of each types of tumor are related to a distinct series of molecular pathogenic defects. Personalized treatment of cancer requires dynamic monitoring the whole abnormal molecular events and interaction among them. MS-based proteomics and pathway network analysis tools have become an essential approach in accelerating personalized treatment. For example, pathway network analysis based on multiple sets of pituitary adenoma proteomics data (DEP data, nitroproteomics data, and protein mapping data) revealed mitochondrial dysfunction, oxidative stress, cell cycle dysregulation, and MAPK-signaling abnormality were significantly associated with pituitary adenoma pathogenesis [172], wich provides new clues to in-depth investigation of pituitary adenoma and discovery of effective biomarkers. Another protein-protein interaction (PPI) analysis of HCCs depicted the molecular portrait and revealed the relationship among metabolism, cytoskeleton biological processes, and HCC metastasis [173].

## Methodology and application of metabolomics in cancer research and clinically relevant outcomes

### Methodology

Metabolism is one of the key components of life. Studies have shown that the physiological state of cells and tissues is determined by both the cell’s regulatory systems and its state of intermediary metabolism [174]. Metabolites are small molecules (< 1 KDa) derived from metabolism, and provide functional information that cannot be directly obtained from genome and proteome of the cellular and tissue states [175, 176]. These metabolic profiles are associated with totally biochemical processes as beginning, intermediate, or end products and provide information on complex interactions between genes and environment of a given condition [177, 178]. Also, metabolites can feed back on other physiological and pathological processes [179–182]. Metabolome contains all endogenous metabolites and is divided into primary metabolome (governed by the host genome) and co-metabolome (dependent on the microbiome) [175]. Metabolome-wide association is able to uncover the etiology decided by the intricate interaction of genes, environment and lifestyles in the general population [183]. Metabolomics is the methodology and theory to comprehensively and dynamically study metabolome [184], including identification biochemical and molecular characteristics of metabolome, characterization of interactions among different metabolites or between metabolites and genetic/environmental factors, and evaluation of biochemical mechanisms related to a given condition such as different pathophysiological processes [185]. In general, metabolomics can be divided into targeted metabolomics and untargeted metabolomics. Targeted metabolomics refers to a method where a specified list of metabolites is measured, typically focusing on one or more related pathways of interest. Targeted metabolomics is commonly driven by a specific biochemical question or hypothesis that motivates the investigation of a particular pathway [176]. Untargeted metabolomics is a globally and simultaneously measurement of as many metabolites as possible from biological samples without bias [176].

NMR spectroscopy (mostly proton NMR, ^1^H-NMR) and chromatography coupled to MS (LC-MS and GC-MS) are two leading spectroscopic techniques used in metabolomics [186]. Numerous favorable characteristics make NMR a beneficial tool in metabolomics research. NMR-based methods have high reproducibility in the laboratory and between laboratories [187–189]. NMR enables the identity of structures for unknown metabolites [190–192] and possesses the ability to non-constructively analyze samples that do not need to separate and elaborately prepare samples, which could be analyzed subsequently with other platforms [193–196]. Moreover, with isotope labeling, NMR provides a window to observe the dynamic changes of metabolite formation and metabolic pathways, which could be used to follow the perturbation of metabolites before and after intervention treatment [197, 198]. Since the 1970s, chromatographic methods have been used to separate complex mixture of metabolites and improve analysis and identification [199]. GC and GC-MS methods have been used to quantify metabolic profiling, but GC-MS is largely limited to volatile compounds [199]. LC-MS has significantly improved the capability of MS-based metabolomics because it is more sensitive than ^1^HNMR and can identify and quantify a few hundred metabolites within a single extract [199, 200]. However, each method has its own advantages and disadvantages. NMR is less sensitive than MS by up to 100-fold, and the instrument is expensive. LC-MS is highly sensitive, but it is necessary to separate and prepare samples, which might potentially modify metabolite structure to increase the difficulty in analysis. None of them alone can effectively identify and quantify, with sufficient sensitivity and precision, the diverse range of metabolites and their dynamic changes in cells. An integrated method of these methods is necessary to increase the accuracy and efficiency of identification of those metabolites and benefit the development of metabolomics [201]. The characteristics of NMR, GC-MS, and LC-MS, and the examples of applications in cancer research were presented (Table 3).

**Table 3:** Summary of metabolomic techniques and examples of their applications in cancer research

Technique	Strengths	Limitations	Related applications in cancer research	Information of samples	Result and significance in PPPM
NMR	Nondestructively analyze samples either in body fluids or in vivo	Low sensitivity	Madhu et al. 2016 [202]	Ten benign prostate tissue samples, seven prostate cancer (PCa) specimens from untreated patients, six PCa specimens from patients treated with Degarelix	This study demonstrated the concentration of specific metabolites could reflect the real-time response of antitumor drug treatment
	High reproducibility and repeatability	Poor quantification ability	Hajduk et al. 2016 [203]	Blood sample form 45 head and neck squamous cell carcinoma patients with radiotherapy (RT) or chemoradiotherapy (CHRT)	This study monitoring the effect of RT based on metabolomics method provide the basis of precision treatment
	Quantification analysis of metabolites	Requires large sample size			
GC-MS	Especially suitable for thermostable and volatile and nonpolar metabolites	Derivatization required, so unfit for polar metabolites such as polyphenos and glycosides			
	High separation efficiency and reproducibility	Extensive sample preparation steps and time consuming	Hadi et al. 2017 [204]	Serum sample from 152 pre-operative breast cancer (BC) patients and 155 healthy controls	This study constructed models using distinct metabolites to diagnose, stage, grade and evaluate neoadjuvant status providing metabolic evidence for early diagnosis and treatment of BC
	Very sensitive	Destructive (sample not recoverable)	Cameron et al. 2016 [205]	Sputum sample from 34 suspected lung cancer (LC) patients, 33 healthy controls	This study demonstrated the feasibility of sputum metabolomics analysis and indicated this method could help ones to noninvasively screen the high-risk population of lung cancer
	High mass accuracy to detect compounds	Derived samples can only be stored for 2-3 days			
	Highly developed compound libraries and software for metabolite identification	Novel compound identification is difficult			
	Can be mostly automated	Cannot be used in imaging			
LC-MS	Be capable to detect the largest potion of metabolome	Lower separation power and reproducibility than GC-MS	Di Gangi et al. 2016 [206]	Serum sample from 40 suspected pancreatic cancer patients and 40 healthy controls	This research identified several metabolites as highly discriminative potential prognostic markers
	Excellent sensitivity	Destructive to samples	Hou et al. 2014 [207]	Plasma from 38 cervical cancer patients with different response to neoadjuvant chemotherapy (NACT)	A prediction model with an AUC of 0.9407 can be used to predict the patient’s response to NACT, which has important implications in personalized treatment and outcomes
	Simple sample preparation and short separation time	Not very been quantified	Mathé et al. 2014 [208]	Urine collected from 469 patients with lung cancer and 536 population controls	Creatine riboside and N-acetylneuraminic acid can be regarded as novel noninvasive biomarkers for the early diagnosis and prognosis of lung cancer
	Detects a wider range of metabolites than GC-MS	High instrumental cost			
	Analysis of more polar compounds without derivatization and ideal for nonvolatile compounds	More instrumental variables than in NMR and GC-MS			

### Application

Cancer is involved in a range of metabolic process changes. Metabolites are the products of the interactions between genes and environment. The metabolites are closer to the phenotype of the organism than genes and proteins. Early diagnosis is critical to improve the survival of cancer patients. Metabolomics is considered as a relatively rapid, accurate and noninvasive method, it is becoming an increasingly popular tool in discovery of diagnostic biomarkers of cancers [209, 210]. Many enthusiastic metabolomic markers have been reported for diagnosis and prognosis in lung cancer [205, 208, 211], breast cancer [204, 212], pancreatic cancer [206], Pca [213–215], bladder cancer [216–218], and epithelial ovarian cancer [219, 220].

For example, metabolomics has been used to discover noninvasive diagnostic biomarkers for lung cancer with high incidence and mortality. The unbiased LC-MS analysis of the metabolic profiling of urines from 469 lung cancer patients and 536 controls [208] revealed creatine riboside and N-acetylneuraminic acid (NANA) were the powerful urinary clinical metabolomic biomarkers for putative diagnosis and prognosis, which was further confirmed in an independent population with 80 patients and 78 controls. Also, sweat metabolomics was used to discover noninvasive biomarkers for diagnosis and prognosis of cancers. LC-MS analysis of metabolome of lung cancers relative to normal smokers identified trisaccharide phosphate as an individual metabolite biomarker to discriminate lung cancer from controls with the specificity of 80% and sensitivity of 72.7% [211], and a panel of five metabolites (trihexose, tetrahexose, suberic acid, monoglyceride MG (22:2), and nonanedioic acid) significantly improved the specificity (80%) and sensitivity (79%). Moreover, the sputum metabolomics analysis [205] between 34 lung cancer patients and 33 healthy controls found that ganglioside GM1 might be a reliable candidate for biomarker and showed that sputum metabolomics method could help ones to screen the high-risk population of lung cancer.

Metabolomics has also been used in breast cancer research. UPLC-MS/MS analysis of saliva metabolite profiling of breast cancer patients identified the ratios of polyamines, eight polyamines, as noninvasive diagnostic biomarker to effectively discriminate breast cancer patients from healthy controls [212]. GC-MS analysis [204] of serum metabolomes of 152 pre-operative breast cancer patients and 155 healthy controls identified seven metabolites (tetradecane, alpha-D-glucopyranoside, methylstearate, dodecane, 1-4-benzene, D-galactose, and octadecanoic acid) that were significantly associated with breast cancers, found metabolic content differs between cancer and benign tissues, and also identified differentiated metabolites for grading, staging and determination of neoadjuvant status.

MS-based metabolomics [206] revealed four metabolites (oleanoic acid, taurochenodeoxycholate, palmitic acid, and d-sphingosine) as highly discriminative potential prognostic markers for pancreatic cancer, a poor prognostic cancer with 5-year survival rate < 5%, demonstrated that palmitic acid has a better discriminating ability compared to the CA19-9 that is only biomarker routinely used for the clinical management of pancreatic cancer, and recommended simultaneous assessment of palmitic acid and CA19-9 to reduce false positives and improve prognosis of patients. It suggests metabolomics plays an important role in prognosis research of pancreatic cancer.

The increase of efficiency and decrease of the side effects in cancer therapy have always been the focus of cancer research, which is actually consistent with the goal of precise medicine that is to use advanced multiomics testing to customize a personalized medical treatment according to their specific biomarker profiling. Cancer genomic profiling is now routinely used to guide the cancer precision medicine, and made some achievements. However, the heterogeneities of cancer tissues and cancer genomes make it impossible alone to guide precise treatment of cancer. Genomic profiling is a powerful tool to provide the information what will happen in tumor, whereas metabolomics can provide the information what has happened and is happening in cancer. Metabolomics has the ability to measure the sum of all these genotypic, environmental and physiological effects, thus it is a very promising method for the use of metabolomics to predict and assess responses to anticancer treatments in cancer research, and it is possible for the use of metabolic profiles to predict the response of individual patients to a class of treatments.

For example, the untargeted serum metabolomics of lung adenocarcinoma patients before chemotherapy identified and constructed a metabolite pattern model to predict the response of pemetrexed and platinum treatment demonstrating the metabolomics-based method is an effective approach to identify appropriate patients who are more likely to a special treatment [221]. Metabolomics analysis of human xenograft model of gastric cancer established a prediction model containing 1-acyl-lysophosphatidycholines, polyunsaturated fatty acids and their derivatives, which can predict the chemosensitivity of cisplatin plus 5-fluorouracil with an accuracy of 90.4% [178]. Similar metabolomics-based predictive studies were also carried out in other types of cancers [209, 219, 220]. Those examples clearly demonstrated that metabolomics is an effective method to stratify patients, establish reliable predictive models to predict the response of cancer patients before the treatment, and improve the efficacy and survival time of patients. Moreover, the immediately measurable metabolic perturbations are occurring in a large number of tissues after exposure to a particular antitumor agent, these metabolic changes represent a biomarker of efficacy or toxicity, which is easily detected by metabolomics methods. A ^1^H MRS-based metabolomics analysis of Degarelix that decreases serum androgen levels in human advanced Pca found that the degree of concentration decline of two metabolites (lactate and t-choline) was able to monitor noninvasively the response of castration [202]. The use of hyperpolarized MRI-based metabolomics to study of targeting PI3K/mTOR pathway in sarcomas found lactate was a biomarker to assess the treatment response to rapamycin [222]. Metabolomics also plays important roles in monitoring radiotherapy toxicity. The ^1^H NMR-based serum metabolomics analysis found the increased N-acetyl-glycoprotein and acetate was the biomarkers to reflect the acute radiation sequelae (ARS) in head and neck squamous cell carcinoma patients [203].

Those evidences clearly demonstrate that metabolomics method is more accurate and faster in assessment of treatment response compared to the traditional method such as imaging examination in evaluation of anticancer effects.

Currently the understanding of cancer is gradually shifted from a genetic disease to a metabolic disorder [223, 224] because metabolites not only reflect the metabolic state of cancer but also feedback the information on the occurrence, development, and consequence of cancer. With the extensive application of metabolomics technology in cancer research, a new term “oncometabolites” are proposed and defined as endogenous metabolites and their accumulation that initiates or sustains growth and metastasis of cancer [225]. A series of oncometabolites have been identified, including 2-hydroxyglutarate and glucose in gliomas and acute myeloid leukemia [226–228], fumarate in papillary kidney cancer [229], succinate in pheochromocytoma [230], sarcosine and choline in Pca [231, 232], glutamine in pancreatic [233, 234], asparagine in ovarian cancer [235], and lactate in breast cancer [236, 237]. Those oncometabolites are leading to identity of novel drug targets and therapeutics.

For example, isocitrate dehydrogenase 1 and 2 (IDH1 and IDH2) are critical metabolic enzymes that catalyze isocitrate to α-ketoglutarate. Mutated IDH1/2 was found a neomorphic enzymatic activity to catalyze α-ketoglutarate to (R)2-hydroglutarate [(R)2-HG] in gliomas [238, 239]. The accumulation of 2-HG inhibits 2-oxoglutarate-dependent oxygenases [240], impairs histone demethylation [241], blocks cell differentiation [242], and promotes tumorigenesis [243]. Tumor with IDH mutation constructs a distinct clinical subset in both leukemia and gliomas. IDH mutations were also identified in multiple cancers, including chondrosarcoma [244], sarcoma [245], and cholangiocarcinoma [246]. IDH mutants become promising candidates of therapeutic targets. A selective R 132H-IDH1 inhibitor (AGI-5198) demonstrated that mIDH1 inhibitor was able to block the production of R-2HG, and induce demethylation of histone and the expression of gliogenic differentiation associated genes, but it did not influence the functions of IDH1 wild-type in a glioma [247]. This inhibitor AGI-5198 also demonstrated the similar effects in human chondrosarcoma cells [248]. The IDH2 inhibitor AGI-6780 also induced differentiation of TF-1 erythroleukemia and primary human acute myelogenous leukemia cells [249]. More and more IDH inhibitors are being developed such as AG-120 [250] and AG-221 [251, 252] in cancers. Those studies clearly indicated that IDH mutations are targetable by small molecules, which provides a promising cancer therapeutic strategy, namely inducible differentiation therapy [253]. Inducible differentiation therapy is to reactivate endogenous differentiation programs, elicit tumor cell maturation, and transit cancer to normal tissue without cytotoxic effects, which can overcome drawbacks of traditional cytotoxic chemotherapy that is to inhibit and kill tumor cells with serious side effects [254]. The initial clinical application of IDH inhibitors, inducible differentiation agents, has demonstrated the strong potential in cancer therapy with minimal toxicity.

Therefore, those oncometabolites, IDH inhibitors and their clinical applications are the strong evidences in support of the importance of metabolomics technology in discovery of new anticancer drugs and therapeutics.

## Methodology and application of radiomics in cancer research and clinically relevant outcomes

### Methodology

Medical imaging technologies such as CT, PET/CT, and MRI play an irreplaceable role in the diagnosis and prognosis of tumors. In general, medical images are regarded as pictures. Physicians visually interpreted these “pictures” solely and draw qualitative and preliminary quantitative conclusions of tumors, including the location of tumor, internal heterogeneity, the overall and marginal morphology of the lesion, the relationship with surrounding tissues, rough measurements of diameter, the volume of tumor, CT and PET/CT values, MRI signal height and other values. This type of information is crucial for the diagnosis of tumors, but it does not accurately reflect the morphological and behavioral complexities of a tumor, with limited benefits in the judgment of treatment sensitivity and prognosis [255]. Whether one could further exploit the medical imaging to obtain the broader characteristics of tumor? In the past decade, medical imaging analysis and recognition technology has developed rapidly [256], which made it possible to extract and quantitatively analyze the entire information and spawned a new discipline-radiomics [257]. Radiomics, based on computer-aided diagnosis and detection systems, is defined as high-throughput extraction and conversion of quantitative features from medical imaging into mineable data and applied the analysis of these data within clinical decision support systems [256–258]. Since medical imaging is routinely used in clinical decision, radiomics, extending the imaging analysis from qualitative to quantitative and finding the clinical significance that cannot be found with the naked eye, may have a clinical impact on cancer research.

**Fig. 4:**
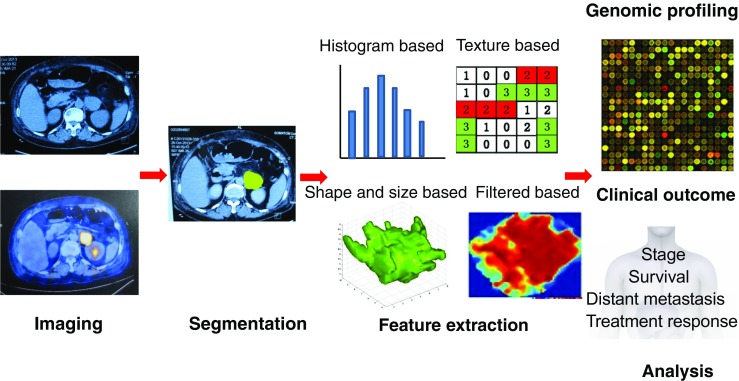
The general workflow of radiomics

The general workflow of radiomics includes 4 steps (Fig. 4): (a) acquisition of high quality and standardized imaging, (b) identification of volumes of interest (VOI) and segmentation, (c) feature extraction and qualification, and (d) analysis and modeling. High quality and standardized imaging is the basic of radiomics. Unlike qualitative analysis, variations in acquisition and image reconstruction will jeopardize the ability to detect biological differences. So standardized imaging is important to eliminate unnecessary confounding variability. Segmentation determines which voxels within an image are analyzed, so it is the most critical and challenging component of radiomics. The ideal segmentation method should provide accurate and reproducible boundaries and should be time efficient, which means the entire process should be as automated as possible with minimum operator interaction. Myriad imaging features can be extracted and divided into tumor intensity histogram-based features, shape-based features, and texture-based features. Only those task-specific features have been selected and analyzed. Ideally, the final model based on selected features and methodology must be internally and externally validated.

### Application

Radiomics, like the other omics, has equivalent potential role in PPPM of cancer. Several studies suggested the potential associations between certain radiomics features and tumor phenotypic patterns [259–261]. Analysis of radiomics-based features, comprehensive quantification information relating to the tumor phenotypes could be obtained [262, 263]. Moreover, potential noninvasive imaging biomarkers for prediction of treatment response and outcomes could also be provided. For example,a PET/CT imaging study in NSCLC showed that abnormal texture as measured by coarseness, contrast, and busyness is associated with nonresponse to chemoradiotherapy and with poorer prognosis [264]. Another study exploring a set of 635 CT-derived imaging features, including intensity, shape, texture, Laplacian of Gaussian, and wavelet filters, found that 35 and 12 features were related to distant metastasis and survival, respectively [265]. The utility of MRI texture features in glioblastoma demonstrated good performance (area under ROC curve > 0.7) in distinguishing different molecular subtypes and predicting 12-month overall survival status (area under ROC curve = 0.69) [266]. Similarly, based on a series of MRI imaging features of 81 patients, a prognostic model was established that has a potential role in guiding personalized treatment selection [267]. In Pca, Haralick texture analysis of prostate MRI has the ability to detect the tumor lesions and differentiating Pca with different Gleson scores [268]. Another study assessed T2-weighted MRI-derived textural features demonstrated that these features corrected significantly with Gleason score and could distinguish Gleason score 3+4 from 4+3 cancers with high sensitive to the pathological difference [269]. There are similar researches in esophageal cancer [270, 271], rectal cancer [272], breast cancer [273, 274] and head and neck cancer [275, 276]. In addition, radiomics could be used to predict radiotherapy-related side effect and guide personalized radiotherapy treatment. For example, the intensity and textural features based on CT of pre- and post-radiation therapy was analyzed in the study of the relationship between radiation dose and the development of radiation pneumonitis. As a result, 12 features showed a significant correlation with pneumonitis [270]. A similar study also found that texture features extracted from CT of nasopharyngeal cancer could be used in predicting parotid shrinkage at the end radiation therapy.

Furthermore, radiomics has distinct characteristics. In the era of precision medicine, genotype of tumor is an important basis for personalized treatment. Due to the high heterogeneity of tumor, the genomic profiling obtained from clinical biopsy is insufficient to reflect the real genomic state of a tumor. Simultaneously, not all cancer patients can undergo biopsy that may induce serious complications. In contrast, almost every cancer patient has radiologic images and radiomics could objectively and precisely provide detailed quantitative features of intra- and intertumoural heterogeneity in a non-invasive manner. Based on the hypothesis that genotypic variation is the source of a proportion of radiomic features variance, a new interdisciplinary radiogenomics mining of radiomics data to detect correlations with genomic patterns has been proposed. Radiogenomics facilitates an in-depth understanding of tumor biology and captures the intrinsic tumor heterogeneity and could provide diagnostic and prognostic imaging biomarkers to guide the precisely personalized treatment [277, 278]. For example, a study of 10 glioblastoma MRI features discovered that the ratio of enhancing to nonenhancing volume was correlated with EGFR overexpression. The enhancing phenotype was correlated with angiogenesis and tumor hypoxia-related genes [259]. Another glioblastoma study based on MRI-derived tumor imaging features demonstrated that TP53 mutant tumors had smaller enhancing and necrotic volumes (*p* = 0.012 and 0.017, respectively) and RB1 mutant tumors had smaller edema volumes (*p* = 0.015) [279]. A study of HCC found that microvascular invasion (MVI), an independent predictor of poor outcomes that cannot be adequately determined before operation, has very important clinical decision significance. In a study of contrast-enhanced computered tomography features of 157 HCC patients, venous invasiveness based on three features (internal arteries, hypodense halo and tumor-liver difference) was identified as a radiogenomic biomarker of MVI derived from a 91-gene HCC “venous invasion” gene expression signature. This biomarker has a good performance in detecting MVI with diagnostic accuracy of 89%, sensitivity of 76%, and specificity of 94%, respectively. Patients with a positive RVI score were associated with low overall survival than patients with negative RVI score in the overall cohort [280]. A study of cholangiocarcinoma in exploring of the relationship between imaging feature and hypoxia markers suggested that both qualitative and quantitative imaging features (based on texture analysis of CT) were correlated with a few hypoxia markers, such as VEGF, EGFR, and CD24 [281]. A study of breast cancer by combining radiogenomics with RNA-seq identified the enhancing rim fraction score, a quantitative dynamic contrast material-enhanced MR imaging IncRNA radiogenomic biomarker, which was associated with metastasis and expression of the known predictor of metastatic progression, HOTAIR [282]. Another potential advantage of radiomics is to identify breast cancer molecular subtypes that are crucial in personalized treatment and no low-cost genetic testing is readily available. For example, a multivariate analysis of relationship between 56 routine MRI-based imaging features (including morphologic, texture, and dynamic features) and molecular subtype demonstrated a strong association between the collective imaging features and both luminal A and Iuminal B molecular breast cancer subtypes. No association was found for either HER2 or basal molecular subtype and the imaging features [283]. Similarly, using the computer-extracted MRI image-based features of 91 biopsy-proven invasive breast cancers from TCGA/TCIA, a classifier model was established and evaluated with receiver operating characteristic analysis, which shown the ability to distinguish between molecular prognostic indicators. This study shows promise for high-throughput discrimination of breast cancer subtypes and may yield a quantitatively predictive signature of advancing precision medicine [284].

## The integration of multi-omics data in cancer research and clinically relevant outcomes

Cancer is a complex disease and involves deregulation in different levels of DNA, RNA, protein, and metabolite; and those different levels of molecules are mutually associated [19, 22, 23, 116]. Any individual study in a different level is insufficient to clarify the intricate pathogenesis of a cancer. Integration of multiple omics data is essential to cancer research and fits the reality of a cancer [19], which will provide a holistic view of what really happened during normal cell malignant transformation and tumor progression, and have the potential in improvement of targeted therapy and the effectiveness of traditional therapies, in clarification of molecular mechanisms of cancer therapeutic resistance, and in discovery of novel biomarkers and targeted drugs.

Integrated omics has been widely used in cancer research. For example, an integrated analysis of genomic and transcriptomic data and long-term clinical outcomes analyzing the changes of gene expression based on somatic gene copy number aberrations revealed some potentially important targeted therapeutic response-related events and proposed a new molecular classification of breast cancer patients [285]. Another integrative analysis of genomic and proteomic data demonstrated that PI3K pathway aberrations are particularly common in hormone receptor-positive breast cancer, which might be important in clinical selection of targeted therapies [286]. The integrated analysis of tissue transcriptomics and urine metabolomics identified four urinary biomarkers that are more credible compared to biomarkers derived from single omics [287]. The integrative analysis of transcriptomics, proteomics, and clinical outcome in HER2-positive breast cancers who acquired resistance to lapatinib revealed EGFR/HER2 signaling was still blocked, and the blocked intensity was weakened by the upregulation of glucose metabolism and endoplasmic reticulum stress pathways [288]. An integral analysis of transcriptomic and proteomic data in glioblatomas revealed a highly significant enrichment of gonadotropin-releasing hormone (GnRH) signaling pathway that was not deciphered with single omics datasets, which demonstrated the promise of multi-omics research and analyses to better understand complex cancers [289]. Moreover, an integrated quantitative proteomics and phosphoproteomics analysis was also used in sorafenib-treated failure HCCs and revealed that this targeted drug can indeed effectively inhibit its target kinase in Raf-Erk-Rsk pathway, but the downstream targets of Rsk-2 (eIF4B, filamin-A and so on) were not influenced, which suggests another alternative pathways might have been active and contribute to the treatment failure [290].

## Outlook

The development of multiomics technologies benefits in-depth understanding of tumor biology. However, it is still very challenging in translating those multiomics techniques into patient and healthcare. These benefits include short-term and long-term benefits. Multiomics approaches have provided a large number of potential biomarkers and targets, which have produced short-term benefits with clear examples described above. Nevertheless, it will take a long time to fulfill the long-term benefits such as sensitive early diagnosis and significantly improved overall survival.

Multiomics technologies have generated an enormous amount of information critical to expanding our understanding of cancer biology and benefited the treatment of tumor patients. For example, in addition to analyzing tissue biopsy, whole genome sequencing could also be used in the circulation of cancer patients. Several studies have demonstrated the ability of whole genome sequencing in detecting chromosomal copy number changes, rearrangements, DNA hypomethylation, SNP and tumor heterogeneity [291–293]. This approach represents a useful method for noninvasive dynamic detection and monitoring of human tumors that is not dependent on the availability of tumor biopsies, which will bring benefits to patients who do not fit to biopsy. NGS benefits greatly to patients with rare cancers and cancer of unknown primary site, for detailed genomic profiling could be used to identify the main drivers of malignant transformation and to cover the shortage of diagnosis and treatment strategies [294, 295]. Linking genomic and proteomic data for biomarker and therapeutic target at the protein levels accelerate the drug development and benefit special subgroups of cancer patients [296]. Recent years, many novel targeted drugs have been developed and their clinical outcomes have been evaluated. Imatinib mesylate is highly efficacious in chronic myeloid leukemias and gastrointestinal stromal tumors [297, 298]. Non-squamous NSCLC patients with EGFR mutation benefited from gefitinib and afatinib with increased tumor response rate and prolonged progression-free survival compared to cytotoxic chemotherapy [299], while sorafenib may derive clinical benefit to NSCLC patients with wild-type EGFR [300]. Although a series of potential biomarkers generated by proteomics, metabolomics, and radiomics have not been approved in the clinical application, some of these candidates (such as AFP-L3 and des-γ-carboxyprothrombin in HCC [156, 301, 302], and sarcosine in Pca [232]) show better sensitivity and specificity compared to the FDA-approved biomarkers. More cancer patients will benefit from these biomarkers, if these biomarkers be validated in follow-up studies.

## Conclusions and expert recommendations

The development of high-throughput and cost-effective multiple omics technologies have extensively used in in-depth understanding of the initiation, progression, and efficacious treatment of a cancer. DNA sequencing technologies, especially the NGS technologies, can detect a more comprehensive character of each major alternation in cancer genome. RNA-seq is a powerful tool to analyze gene expression profiles, and discovers novel intragenic fusion, somatic nucleotide mutations, transcripts, alternative splice forms, and non-coding RNAs. This genome profiling has the potential role in establishing different molecular subtypes and stratification of different patients, which is crucial in precisely personalized treatment. DNA and RNA are vectors of genetic information, and could reflect what will happen in the cells. Proteins encoded by the genes are ultimately the functional performer and could reflect what is really happening in real time or has happen in a given condition. MS-based proteomics demonstrate the powerful role in discovery of new biomarkers, driver events, and personalized therapeutic target, with access to a wide range of protein information from tissues and body fluids of cancer patients. Metabolomics not only provides results from complex gene-environment interactions under any conditions but also can feedback information on physiological and pathological processes. NMR- and MS-based metabolomics can effectively address scientific problems of a cancer, and have made obviously achievements in cancer diagnosis, assessment of response to traditional therapy, and discovery of novel drugs and therapeutics. Radiomics is the bridge between medical imaging and personalized medicine and could objectively and precisely provide detailed quantitative features of intratumoural and intertumoural heterogeneity in a non-invasive manner. Moreover, cancer is essentially a complex disease. Integrative multi-omics data provide a holistic view of the complexity in tumorigenesis, and benefit selection of right patients for targeted therapies and evaluation of traditional treatment strategies for improvement of its therapeutic effects. The multi-omics technologies have make significant achievements in cancer research and clinically relevant outcomes, and will surely accelerate the cancer research with the breakthrough of technical limitations and ultimately benefit more cancer patients in the world.

We recommend this review article to promote the education program regarding the roles of multi-omics in cancer research and clinically relevant outcomes, and emphasize the scientific importance of multi-omics in PPPM in a cancer, especially in discovery of multi-omics-based biomarkers for predictive diagnosis and prognosis assessment of a cancer, and in systematical clarification of molecular mechanisms to discover effectively therapeutic targets for a cancer.

## Abbreviations

CAGE: Cap analysis of gene expression
CID: Collision induced dissociation
CML: Chronic myelogenous leukemia
ECD: Electro capture dissociation
ETD: Electron transfer dissociation
FTICR: Fourier transform ion cyclotron resonance
GC: Gas chromatography
HCC: Hepatocellular carcinoma
HP: Fucosylated haptoglobin
HPLC: High performance liquid chromatography
IARC: International Agency for Research on Cancer
ICGC: International Cancer Genome Consortium
lncRNAs: Long ncRNAs
LC: Liquid chromatography
LSCC: Lung squamous cell carcinoma
MALDI: Matrix-assisted laser desorption ionization
MDLC: Multi-dimensional LC
MPSS: Massively parallel signature sequencing
MRM: Multiple reaction monitoring
MS: Mass spectrometry
MS/MS: Tandem mass spectrometry
NANA: N-acetylneuraminic acid
ncRNAs: Non-coding RNAs
NGS: Next-generation sequencing
NMR: Nuclear magnetic resonance
NSCLC: Non-small cell lung cancer
PPPM: Predictive, preventive, and personalized medicine
SAGE: Serial analysis of gene expression
SCLC: Small cell lung cancer
SCO: Small cell osteosarcoma
SRM: Selected reaction monitoring
SWATH: Sequential window acquisition of all theoretical mass spectra
TCGA: Cancer Genome Atlas
TOF: Time-of-flight
1DGE: One-dimensional gel electrophoresis
2DGE: Two-dimensional gel electrophoresis
2D-DIGE: Two-dimensional difference in-gel electrophoresis
WGS: Whole genome sequencing
